# A Multi-Modal Gait Database of Natural Everyday-Walk in an Urban Environment

**DOI:** 10.1038/s41597-022-01580-3

**Published:** 2022-08-03

**Authors:** Viktor Losing, Martina Hasenjäger

**Affiliations:** Honda Research Institute Europe GmbH, Offenbach, 63073 Germany

**Keywords:** Databases, Visual system, Neuroscience

## Abstract

Human gait data have traditionally been recorded in controlled laboratory environments focusing on single aspects in isolation. In contrast, the database presented here provides recordings of everyday walk scenarios in a natural urban environment, including synchronized IMU−, FSR−, and gaze data. Twenty healthy participants (five females, fifteen males, between 18 and 69 years old, 178.5 ± 7.64 cm, 72.9 ± 8.7 kg) wore a full-body Lycra suit with 17 IMU sensors, insoles with eight pressure sensing cells per foot, and a mobile eye tracker. They completed three different walk courses, where each trial consisted of several minutes of walking, including a variety of common elements such as ramps, stairs, and pavements. The data is annotated in detail to enable machine-learning-based analysis and prediction. We anticipate the data set to provide a foundation for research that considers natural everyday walk scenarios with transitional motions and the interaction between gait and gaze during walking.

## Abstract (summary)

**Table:** 

Measurement(s)	Full-body motion data including positional skeleton data, joint angles, acceleration and velocity of 23 body segments • Foot pressure • Eye gaze position including first-person scene video
Technology Type(s)	Xsens body suit with 17 IMU sensors • IEE ActiSense Smart Footwear Sensor with 8 sensor segments per foot • Pupil Invisible Glasses Eyetracker
Independent variable(s)	pressure values per foot • eye gaze X, Y position

## Background & Summary

The scientific assessment and modeling of human locomotion has been a central topic in various domains such as medicine, ergonomics, robotics, and sports^1–8^. Traditionally, human gait data have been recorded in controlled laboratory settings^9–11^, e.g., on catwalks or treadmills. However, based on these data, it is impossible to model human gait in more natural and challenging environments where people exhibit a richer gait behavior. Such models are necessary, e.g., for a satisfactory user experience with assist devices that can be used in a clinical environment and in daily life.

Often gait data are available as part of human activity data sets^12–15^ and hence they rarely contain ground truth information for the segmentation of single steps. These data sets provide lower body IMU data and sometimes include FSR data to provide ground truth for step segmentation. There are only a few dedicated human walk data sets that show walking in natural outdoor environments^16–18^. The focus is on walking speed variation and measurement of different walk patterns in isolation.

We aimed to create a richly annotated gait data set of natural, everyday walk scenarios requiring continuous walking of 5 to 15 minutes that naturally contain diverse gait patterns such as level walking, walking up/down ramps and stairs, as well as the corresponding transitions in between. In particular, the recordings include the natural interaction with other pedestrians and cyclists that affect the subjects’ gait behavior. We provide whole-body data from 17 IMU sensors to enable a wide variety of motion modeling. Additionally, we include plantar foot pressure data that yield accurate foot contact information and may be used independently from the IMU data.

The data set consists of 9 hours of gait data recorded from 20 healthy subjects. They walked across three different courses in a public area around a suburban train station. A single repetition of each course required several minutes of walking and captured many common elements such as straight and curvy passages, slopes, stairs, and pavements. We annotated the walking mode, e.g., regular walk, climb/descend stairs, ascend/descend slopes, interactions with other pedestrians/cyclists, curves and turnarounds, as well as terrain segments. The timings of heel strike and toe-off events are provided as well.

Another unique feature of our data set is the usage of a mobile eye tracker to record the gaze behavior of our participants during walking. Humans extensively use visual information of the environment for strategic control planning^19^. For instance, they adapt their gait speed and gaze angle to the complexity of the environment^20^. Spatio-temporal visual information is essential for proper foot positioning on complex surfaces^21^. Thus gaze may serve as an indicator of human intention during walking^22^ as well as an estimator of fall risk^23–26^. To our knowledge, this data set is the first publicly available database that provides gait motion data together with the corresponding visual behavior. In particular, the data allows the estimation of the gaze trajectory by combining the gaze position with the head orientation. As gaze is known to be an early predictor of human intention^27,28^, we think that the analysis of gaze patterns as a predictive signal for the anticipation of walk mode transitions provides an exciting research opportunity.

In summary, we anticipate that this data set will provide a foundation for future research exploring machine learning for real-time motion recognition and prediction, potentially incorporating visual behavior and analyzing its benefits.

## Methods

### Participants

**Fig. 1:**
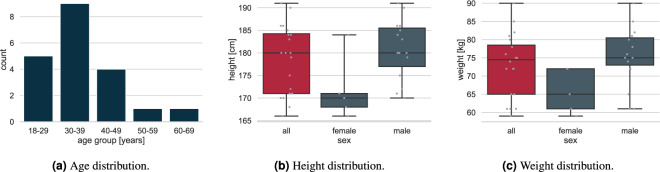
Statistics of participants’ (**a**) age, (**b**) height, and (**c**) weight.

Twenty-five healthy adults with normal or corrected-to-normal vision volunteered to take part in the study. The data from five participants were incomplete due to sensor failures and are not included in the data set. The anthropometry of the remaining 20 participants, 5 females and 15 males, is given in Table 1. The participants’ average height of 178.55 ± 7.6 cm corresponds to the average height in central Europe, while their average weight of 72.95 ± 8.7 kg was slightly below the central European average, i.e. all participants were slim, cf. Figure 1.

**Table 1:** Anthropometry information of the participants.

ID	Age [years]	Sex	Weight [kg]	Height [cm]	Hip Height [cm]	Insole size
1	50–59	female	59	171	91	M
2	30–39	male	74	186	95	L
3	18–29	male	80	185	112	XL
4	18–29	male	78	184	111	XL
5	30–39	male	61	172	98	M
7	18–29	male	82	180	98	L
8	30–39	female	65	168	94	M
10	40–49	male	81	186	102	XL
12	40–49	female	61	166	93	M
13	18–29	male	76	183	92	L
14	30–39	male	90	190	102	XL
15	30–39	female	72	184	101	L
16	30–39	male	61	171	90.5	M
17	40–49	male	72	191	100	XL
18	18–29	male	85	180	93	L
19	60–69	female	72	170	92	M
22	40–49	male	75	175	91	L
23	30–39	male	75	179	93	L
24	30–39	male	65	170	92	L
25	30–39	male	75	180	95	L
Summary	36.8 (±10.75)	5 f, 15 m	72.95 (±8.68)	178.55 (±7.64)	96.77 (±6.29)	—

Body height and hip height include the sole height of the shoes. The hip height was measured from the floor to the greater trochanter and may be considered as leg length in gait analysis.

All participants provided written informed consent, including written permission to publish the data of this study. The study was approved by the Bioethics Committee in Honda’s R&D (97HM-036H, Dec. 14, 2020).

### Experimental tasks

**Fig. 2:**
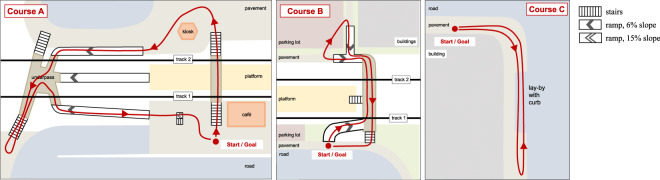
Maps of walking courses A, B, and C.

The participants were asked to complete different walking courses in the area of a suburban train station that included walking on level ground, ascending and descending stairs, walking up and down ramps, and stepping up and down a curb. Figure 2 shows maps of the three walking courses.

**Fig. 3:**
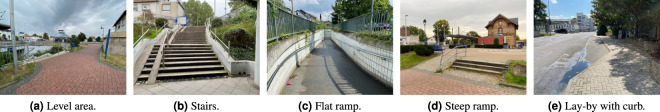
Photos of the experiment location.

Courses A and B include level walking, walking up and down ramps, and up and down stairs. Figure 3(a–d) illustrate the walking tasks A and B: Fig. 3(a) shows a level area. Figure 3(b) shows typical stairs. They consist of one or two groups of 8 to 13 steps that are separated by landings. Typical ramps, as shown in Fig. 3(c), have a slope of 6% and a length of 50 m to 70 m. There is one short and steep ramp in course A as shown in Fig. 3(d). Here the slope is 15%, and the length is approx. 3 m. The walking distance for each course is roughly 500 m.

Course C includes straight level walking, walking a 90-degree curve, stepping up and down a curb in a lay-by, and turning by 180 degrees. The lay-by is shown in Fig. 3(e). The curb height here is 10 cm. The walking distance in course C is roughly 200 m.

### Sensors

**Fig. 4:**
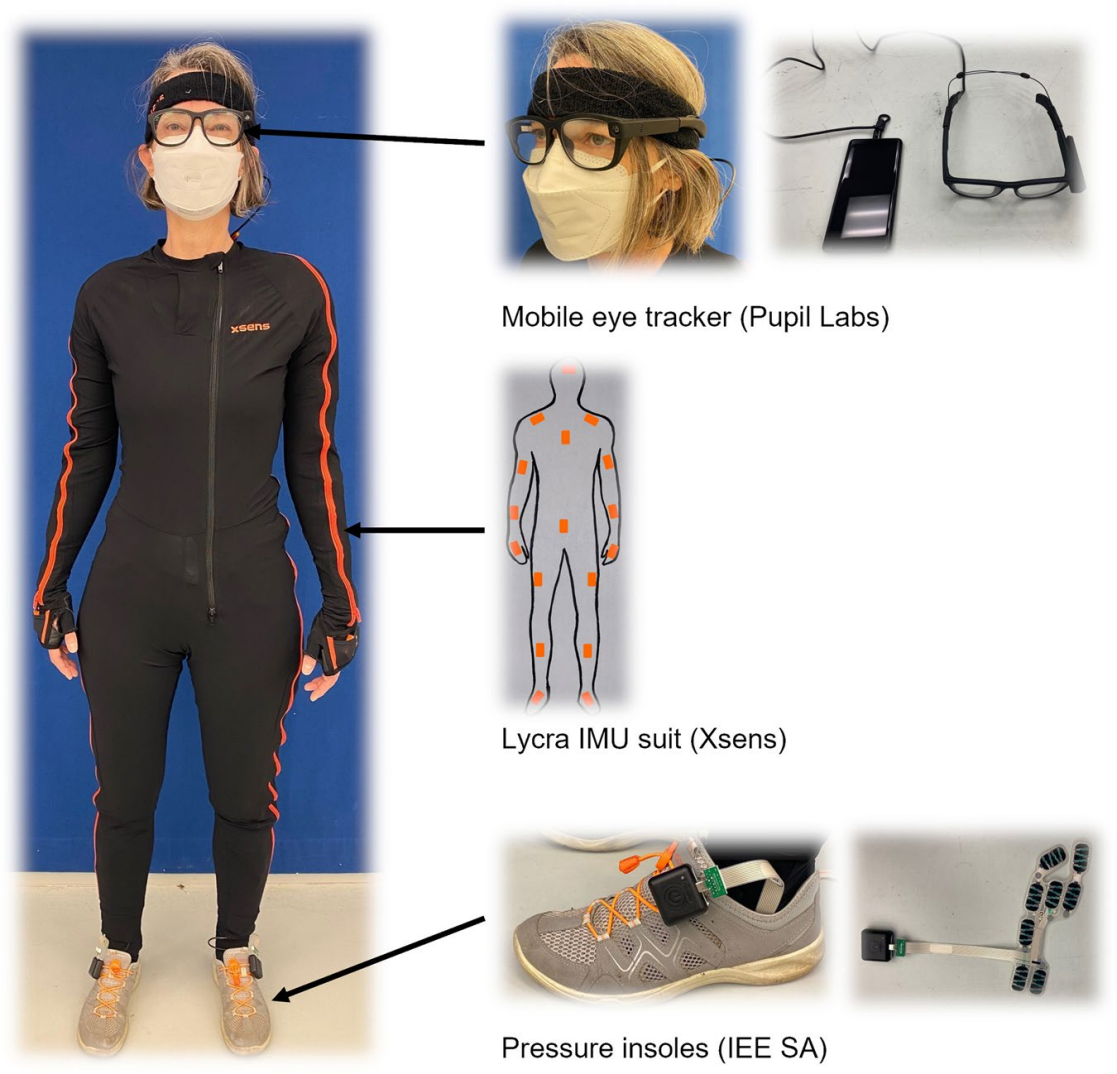
The sensory equipment of the participants (left). They wore a mobile eye tracker, the Pupil Invisible Glasses^31^ (top right), an Xsens full-body motion suit with 17 IMU sensors (middle right), and the IEE ActiSense Smart Footwear Sensor insoles (IEE S.A., Luxembourg) to record foot pressure data (bottom right). Note that the ICUs of the pressure insoles, the small black boxes attached to the shoes, also contain an IMU each. This means that there are two IMUs from different measurement systems attached to each foot: the IMU from the motion capture suit is located below the shoe tongue in the middle of the top of the instep and the pressure insole IMU is located on the shoe on the side of the top of the instep. The participant shown in this figure provided permission for their likeness to be used.

The participants were equipped with the following sensors, cf. Figure 4:

- **Inertial measurement units (IMUs)**. For tracking of motion and posture, we used a full-body inertial kinematic measurement system, the Xsens motion capture suit^29^ consisting of the MVN-Link BIOMECH full-body system and the MVN Link lycra suit. The system consists of 17 IMU sensors with 3D rate gyroscopes for measuring angular velocity, 3D linear accelerometers measuring accelerations including gravitational acceleration, 3D magnetometers for measuring the Earth’s magnetic field, and a barometer to measure the atmospheric pressure. The IMUs are placed on the head, sternum, sacrum, and on the shoulders, upper arms, forearms, hands, upper legs, lower legs, and feet.
- **Force sensitive resistors (FSRs)**. Foot pressure data we recorded using the IEE ActiSense Smart Footwear Sensor insole^30^ (IEE S.A., Luxembourg). The measurement system consists of thin, foil-based, removable pressure insoles with eight high dynamic pressure sensing cells that are inserted into the shoes below the shoes’ insoles. The FSR sensor cells are located below the hallux, the toes, the heads of the first, third, and fifth metatarsal, resp., the arch, and the left and right side of the heel. The pressure insoles are controlled by ECUs that are clipped to the participants’ shoes and come with IMUs consisting of a 3D accelerometer, a 3D gyroscope, and a magnetometer. Note, that here the accelerometer and gyroscope axis coincide while the magnetometer orientation is rotated by 180 degrees around the accelerometer/gyroscope x-axis.
- **Eye tracker**. Eye-tracking data were recorded with a mobile eye tracker, the Pupil Invisible Glasses^31^. The eye tracker is worn like a regular pair of glasses. Two small cameras on the bottom rim of the glasses capture the wearer’s eye movements by using infrared light (IR) LEDs for tracking of the pupil and map the wearer’s gaze point into a scene video captured by a scene camera attached to the spectacle frame.

### Data collection

#### Hardware set-up

The participants were asked to bring tightly fitting clothes and comfortable, flat, lace-up shoes with removable insoles. They wore the Xsens suit over their clothes. The FSR insoles were inserted in their shoes below the shoe insoles. The Xsens suit requires a separate calibration recording before the actual data recording. This calibration consisted of the participant standing in a neutral pose for 5 seconds than walking forward for 5 to 10 meters, making a u-turn, walking back to the starting position, turning and again standing in neutral pose for 5 seconds. The other sensors did not need a calibration procedure. Their proper functioning was checked using their associated smartphone applications. To facilitate the synchronization of the different sensors, we asked the participants to look at their feet for the first few and last few steps of each recording.

#### Experimental tasks

The participants were asked to complete three repetitions of walking courses A and B, resp., and five repetitions of walking course C. They were instructed to walk at their preferred, normal speed and to take a break whenever necessary. All participants completed each walking task without taking a break. One experimenter followed them at a distance to give directions and support the participant if necessary.

The experiments took place in dry weather conditions either in the late morning or the early afternoon to avoid busy commuting times at the train station. However, all participants encountered commuters and passers-by during the experiments so that the data contains side-stepping maneuvers. Note also that some participants chose to take two steps at a time when climbing stairs.

**Fig. 5:**
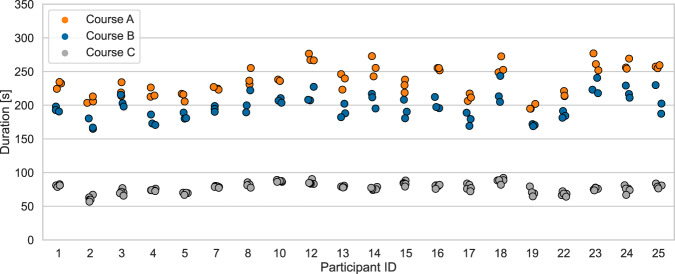
Experiment duration in seconds for each participant and each task.

The average time for completing one repetition of courses A, B, and C was 235 s ± 22 s, 198 ± 19 s, and 77 ± 7 s., resp. Figure 5 illustrates the time it took each participant to complete one repetition of each task. Note that the participant with ID 7 completed six instead of five repetitions of course C. The total recording time of the complete data set amounts to 9:22 hrs with 3:55 hrs for course A, 3:17 hrs for course B, and 2:10 hrs for course C.

### Data processing

The data were recorded on-device and transferred to a desktop computer for post-processing. For each sensor, we used the post-processing software provided by the respective manufacturer.

- **IMU Data**. The Xsens IMU data were recorded with a sampling frequency of 240 Hz. The raw sensor data was post-processed with the Xsens MVN software^29^ (MVN Studio 4.97.1 rev 62391) that computes full-body kinematic data based on a biomechanical model of the participant and sensor fusion algorithms. We provide the full data as post-processed by MVN Studio. Magnetometer data are subject to magnetic distortion from the environment and should be used with care. The resulting data were saved in MVNX file format for further processing.
- **FSR Data**. FSR data were recorded with a sampling frequency of 200 Hz. The IEE ActiSense Smart Footwear Sensor insoles^30^ come with a tool to convert the raw digital values to voltages and to convert the raw accelerometer, gyroscope, and magnetometer data to accelerations, angular rates and magnetic flux density. Additionally, the tool synchronizes the data from both feet. The resulting data is saved in CSV file format.
- **Eye Tracking Data** The gaze data were recorded with a sampling rate of 66 Hz. We used the open-source software Pupil Player^32^ (v3.4) to export the gaze position data to CSV file format and to create a scene video with a gaze position overlay. In a second step, we blurred passers-by and license plates in the resulting scene video for data protection reasons.

**Fig. 6:**
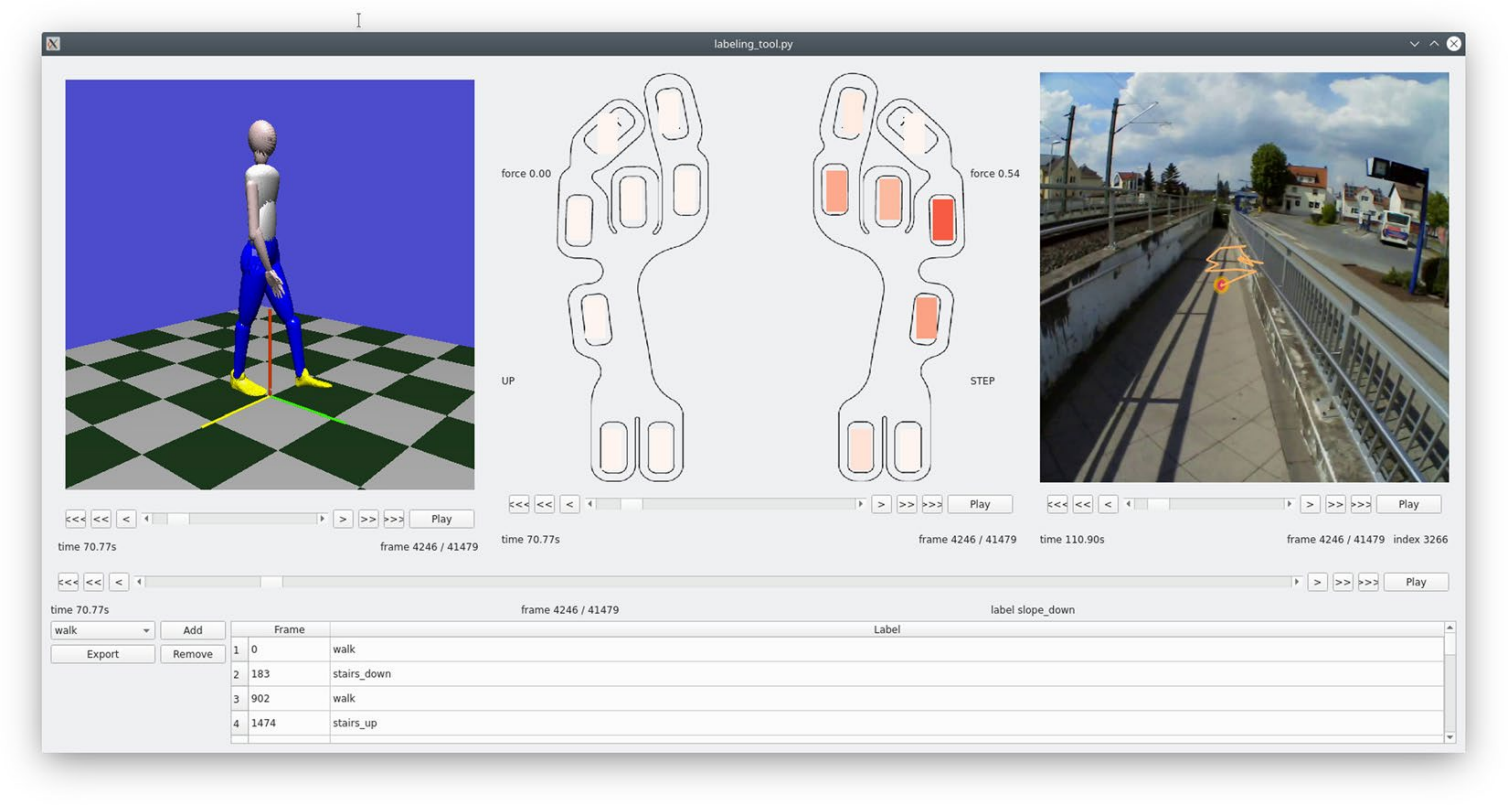
Visualization tool that jointly displays all three sensor modalities. The body posture is based on the XSens segment positions. In the case of the insoles, eight pressure segments are shown for each foot as well as a binary state that indicates whether the foot is on the ground. The scene video including the current fixation as well as the recent gaze trajectory is visualized from the eye tracker recordings.

The data of all three sensors have been down-sampled to 60 Hz. In the case of the IMU data, we kept every fourth data point, whereas we did a linear interpolation of the FSR and eye-tracking data values. All three modalities have been synchronized manually by one experimenter and validated by the other, using the visualization and labeling tool shown in Fig. 6 that is provided with the software related to this data set. The participants were asked to look at their feet during the first and last few steps of each recording to facilitate the post-synchronization. This is required in particular to synchronize the eye tracker recordings with the other two modalities. Some example videos showing all sensor modalities after the synchronization procedure are available in the code repository related to the data set.

Using the same tool, the data was labeled by walk mode and walk orientation: The walk modes are ‘walk’, ‘stairs_down’, ‘stairs_up’, ‘slope_down’, ‘slope_up’, and ‘pavement_up’, ‘pavement_down’ to indicate stepping up or down a curb. The walk orientations are ‘straight’, ‘curve_right’, ‘curve_left’, ‘turn_around_clockwise’, and ‘turn_around_counterclockwise’.

Additionally, we label whether or not the participant interacts with passers-by, i.e. whether or not the participant’s motion trajectory is affected by the motion of other persons in their surroundings. It has been shown that gaze is the main source of information used by pedestrians to control their motion trajectory^33^. Therefore, we annotated encounters with other persons as interaction based on the gaze behavior from the eye tracker that was overlayed over the eye tracker’s world video. We defined an interaction to start as soon as other persons were visually fixated by the participants and to end when the persons left the field of vision.

To easily identify identical course segments over participants and repetitions, the walk courses were segmented by walk mode and consecutively numbered, cf. Figure 8. Since each task was recorded in one go, we included a counter to indicate the repetition of the walking task.

#### Step detection

**Fig. 7:**
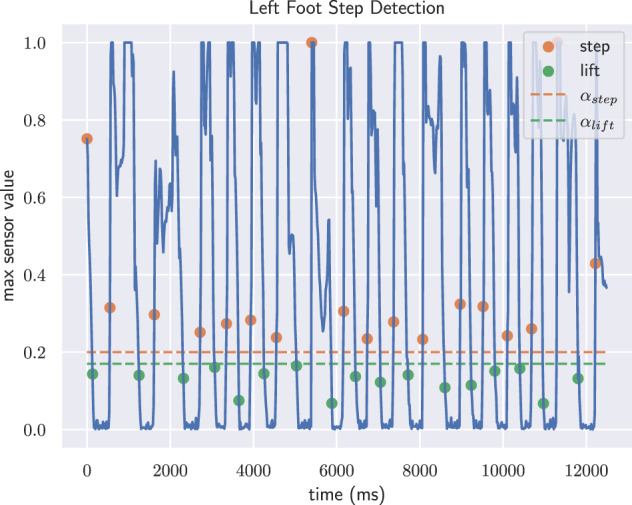
Illustrative example of the heel strike and toe-off detection based on two thresholds.

To simplify gait analysis, we determine the heel strike and toe-off events by the pressure outputs of the insole sensors. Similar to the approach of Hassan *et al*.^34^, we use a heuristic based on two thresholds. We normalize the measured values of each sensor cell for each recording between the first and 99th percentile to remove outliers and achieve an invariance against different body weights, shoe characteristics etc. A foot is assumed to be on the ground if its maximum sensor output surpasses the threshold *α*_step_ and lifted if it falls below the threshold *α*_lift_. We consider all sensor cells because the most relevant ones can vary, particularly for stairs and slopes or when subjects perform evasive motions due to other pedestrians or cyclists. A heel strike or toe-off event is given for the first moment the foot switches from being lifted to being on the ground and vice versa. An illustrative result of the detected events is depicted in Fig. 7.

**Fig. 8:**
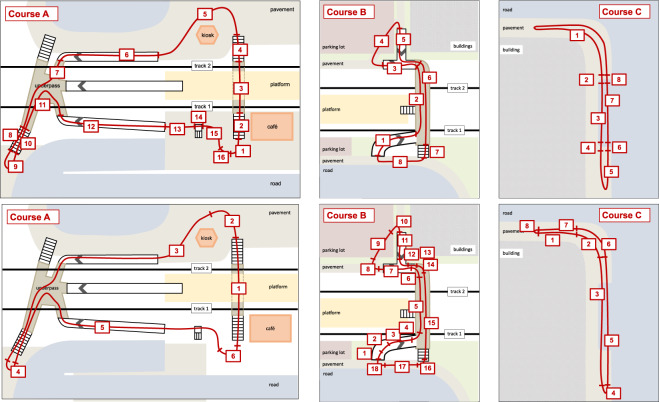
Segments according to walk mode (upper row) and walk orientation (lower row) in walking courses A, B, and C.

Both thresholds *α*_step_ and *α*_lift_ are optimized using a random search on a small set of annotated recordings, minimizing the mean absolute error between the ground truth events and the detected ones. The set of annotated recordings contains five different subjects. For each of those the heel-strike and toe-off events for 60 steps were annotated, thereby solely using the insole visualization shown in Fig. 6. The average temporal difference between detected and annotated events of a test subject is approx. 3.5 ms for heel strikes and approx. 5.5 ms for toe-offs, which is accurate considering the signal frequency of 60 Hz.

We also provide the estimated foot contact data calculated by the Xsens software, however, in our experience they are often inaccurate, particularly for slopes and stairs, and we suggest to treat them with caution.

## Data Records

We provide the data on the figshare data-sharing platform^35^. The repository contains a folder with detailed documentation on the walk courses, including photos to illustrate the area, length, and slope of the ramps and the number, height, and width of the steps in the stairs. The processed data is provided in a file structure that is organized hierarchically by experimental task and participant. Each participant folder contains synchronized CSV data files with (i) eye tracker data (8 columns), (ii) pressure insoles data (91 columns), (iii) full-body Xsens data (757 columns), (iv) labels (22 columns) and (v) the eye tracker scene video in MP4 format. Detailed lists and explanations of all data columns in each file are given in Tables 2–5 and are also provided in the documentation folder on the data-sharing platform. Note that all files contain columns with the experiment time, the participant ID, and the experimental task. The experiment time is synchronized over all sensors and may be used to join data from several files.

**Table 2:** Explanation of data columns in the eye tracker data files.

Column	Unit	Description
time	milliseconds [ms]	experiment time
participant_id	—	unique participant identifier
task	—	experimental task
gaze_timestamp	seconds [s]	timestamp of the source image frame
world_index	—	index of closest world video frame
confidence	—	Assessment by the pupil detector on how sure we can be on this measurement. A value of ‘0’ indicates no confidence. ‘1’ indicates perfect confidence. For useful data, the condifence should be >0.6.
eye_norm_pos_{x | y}	normalized coordinates	{x | y} position in the eye image frame

**Table 3:** Explanation of data columns in the pressure insole data files.

Column	Unit	Description
time	milliseconds [ms]	experiment time
participant_id	—	unique participant identifier
task	—	experimental task
{Left | Right}_Hallux	millibar [mbar]	pressure measured by the {left | right} foot hallux FSR sensor
{Left | Right}_Toes	millibar [mbar]	pressure measured by the {left | right} foot toes FSR sensor
{Left | Right}_Met{1 | 3 | 5}	millibar [mbar]	pressure measured by the {left | right} foot {first | third | fifth} metatarsus FSR sensor
{Left | Right}_Arch	millibar [mbar]	pressure measured by the {left | right} foot arch FSR sensor
{Left | Right}_Heel_{L | R}	millibar [mbar]	pressure measured by the {left | right} foot {left | right} heel FSR sensor
{Left | Right}_Hallux_norm		normalized pressure measured by the {left | right} foot hallux FSR sensor
{Left | Right}_Toes_norm		normalized pressure measured by the {left | right} foot toes FSR sensor
{Left | Right}_Met{1 | 3 | 5}_norm		normalized pressure measured by the {left | right} foot {first | third | fifth} metatarsus FSR sensor
{Left | Right}_Arch_norm		normalized pressure measured by the {left | right} foot arch FSR sensor
{Left | Right}_Heel_{L | R}_norm		normalized pressure measured by the {left | right} foot {left | right} heel FSR sensor
{Left | Right}_Acc_{x | y | z}	g-force (9.806 ms^−2^), [g]	linear acceleration measured by the IMU on the {left | right} foot in {x | y | z}-direction
{Left | Right}_Gyr_{x | y | z}	degrees per second [dps]	angular rate measured by the IMU gyroscope on the {left | right} foot, {x | y | z}-axis
{Left | Right}_Mag_{x | y | z}	microtesla [μT]	magnetic field measured by the IMU magnetometer on the {left | right} foot, {x | y | z}-component
{Left | Right}_Temp	degree Celsius [°C]	temperature measured by the IMU temperature sensor on the {left | right} foot
{Left | Right}_Toes_raw		raw pressure value from the {left | right} foot toes FSR sensor
{Left | Right}_Hallux_raw		raw pressure value from the {left | right} foot hallux FSR sensor
{Left | Right}_Met{1 | 3 | 5}_raw		raw pressure value from the {left | right} foot {first | third | fifth} metatarsus FSR sensor
{Left | Right}_Arch_raw		raw pressure value from the {left | right} foot arch FSR sensor
{Left | Right}_Heel_R_raw		raw pressure value measured by the {left | right} foot right heel FSR sensor
{Left | Right}_Heel_L_raw		raw pressure value measured by the {left | right} foot leftt heel FSR sensor
{Left | Right}_Acc_{x | y | z}_raw		raw linear acceleration value measured by the IMU on the {left | right} foot in {x | y | z}-direction
{Left | Right}_Gyr_{x | y | z}_raw		raw angular rate measured by the IMU gyroscope on the {left | right} foot, {x | y | z}-axis
{Left | Right}_Mag_{x | y | z}_raw		raw magnetic field measured by the IMU magnetometer on the {left | right} foot, {x | y | z}-component
{Left | Right}_Max_Pressure_norm		maximum normalized pressure of the {left | right} foot

**Table 4:** Explanation of data columns in label data files.

Column	Unit	Description	Values
time	milliseconds [ms]	experiment time	
participant_id	none	participant identifier	1, 2, 3, 4, 5, 7, 8, 10, 12, 13, 14, 15, 16, 17, 18, 19, 22, 23, 24, 25
task	—	experimental task	A, B, C
walk_mode	none	label indicating the walk mode	‘walk’, ‘stairs_up’, stairs_down’, ‘slope_up’, ‘slope_down’, ‘pavement_up’, ‘pavement_down’
walk_orientation	none	label indicating the spatial orientation	‘straight’, ‘curve_right’, ‘curve_left’, ‘turn_around_clockwise’, ‘turn_around_counterclockwise’
walk_interaction	none	label indicating whether or not there is an interaction between the participant and passengers	yes or no
terrain	none	label indicating the route section of each task	Courses A and B: 1–16. Course B: 1–8.
repetition	none	repetition counter of the task	Courses A and B: 1–3. Course C: 1–5 (except for subject ID07 who completed 6 repetitions).
insoles_{Left | Right}Foot_is_step	none	heel strike indicator of {left | right} foot determined from pressure insole measurements	True or False
insoles_{Left | Right}Foot_is_lifted	none	toe-off indicator of {left | right} foot determined from pressure insole measurements	True or False
insoles_{Left | Right}Foot_on_ground	none	indicator of {left | right} foot ground contact as determined from pressure insoles	True or False
insoles_{Left | Right}Foot_time_to_step	milliseconds [ms]	time to next heel strike of {left | right} foot	
insoles_{Left | Right}Foot_time_to_lift	milliseconds [ms]	time to next heel strike of {left | right} foot	
xsens_footContacts_{Left | Right}Foot_Heel	none	indicator of {left | right} heel contact with ground determined from xsens data	True or False
xsens_footContacts_{Left | Right}Foot_Toe	none	indicator of {left | right} toe contact with ground determined from Xsens data	True or False

**Table 5:** Explanation of data columns in processed Xsens data files.

Column	Unit	Description
time	ms	experiment time, synchronized over all sensors
participant_id	—	unique participant identifier
task	—	experimental task
orientation_<segment>_{q1 | qi | qj | qk}	—	quaternion orientation of the segment with respect to the global frame.
position_<segment>_{x | y | z}	meter [m]	position of the origin of the segment in the global frame.
velocity _<segment>_{x | y | z}	meter per second [ms^−1^]	velocity of the origin of the segment in the global frame.
acceleration_<segment>_{x | y | z}	meter per second squared [ms^−2^]	acceleration of the origin of the segment in the global frame.
angularVelocity_<segment>_{x | y | z}	radian per second [rads^−1^]	angular velocity of the segment in the global frame.
angularAcceleration_<segment>_{x | y | z}	radian per second squared [rads^−2^]	angular acceleration of the origin of the segment in the global frame.
footContacts_{LeftFoot | RightFoot}_{Heel | Toe}	—	Boolean value defining if contact points were detected for each frame.
sensorFreeAcceleration_<sensor>_{x | y | z}	meter per second squared [ms^−2^]	sensor free acceleration of the sensor.
sensorMagneticField_<sensor>_{x | y | z}	anatomic units [a.u.]	sensor magnetic field of the sensor.
sensorOrientation_<sensor>_{q1 | qi | qj | qk}	—	sensor orientation quaternion of the sensor in the global frame.
jointAngle_j<joint>_{x | y | z}	degree [°]	Euler representation of the joint angle calculated using the Euler sequence ZXY using the ISB based coordinate system.
jointAngleXZY_j<joint>_{x | y | z}	degree [°]	Euler representation of the joint angle calculated using the Euler sequence XZY using the ISB based coordinate system. Note: The joint angle using Euler sequence XZY is calculated and exported for all joints, but commonly only used for the shoulder joints, and it may depend on the movement of the shoulder if it is appropriate to use.
jointAngleErgo_j<joint>_{x | y | z}	degree [°]	Euler representation of the ergonomic joint angles used in ergonomic analysis calculated using the Euler sequence ZXY using the ISB based coordinate system.
jointAngleErgoXZY_j<joint>_{x | y | z}	degree [°]	Euler representation of the ergonomic joint angles used in ergonomic analysis calculated using the Euler sequence XZY using the ISB based coordinate system.
centerOfMass_{x | y | z}	meter [m]	position of the body Center of Mass in the global frame.

Here <segment> is one of the 23 Xsens body segment labels and <joint> is one of the 22 joint labels as listed in Table 6. The variables. The variable names follow the Xsens MVN convention as documented in the MVN User Manual^42^.

**Table 6:** List of Xsens motion sensor locations, segment labels, and joint labels.

	Sensor Location	Segment label	Joint Label
1	pelvis	Pelvis	L5S1
2	T8	L5	L4L3
3	head	L3	L1T12
4	right shoulder	T12	T9T8
5	right upper arm	T8	T1C7
6	right forearm	Neck	C1Head
7	right hand	Head	RightC7Shoulder
8	left shoulder	RightShoulder	RightShoulder
9	left upper arm	RightUpperArm	RightElbow
10	left forearm	RightForeArm	RightWrist
11	left hand	RightHand	LeftC7Shoulder
12	right upper leg	LeftShoulder	LeftShoulder
13	right lower leg	LeftUpperArm	LeftElbow
14	right foot	LeftForeArm	LeftWrist
15	left upper leg	LeftHand	RightHip
16	left lower leg	RightUpperLeg	RightKnee
17	left foot	RightLowerLeg	RightAnkle
18		RightFoot	RightBallFoot
19		RightToe	LeftHip
20		LeftUpperLeg	LeftKnee
21		LeftLowerLeg	LeftAnkle
22		LeftFoot	LeftBallFoot
23		LeftToe	

## Related Data Sets

An overview of related data sets is given by Table 7. Our database differs from the available ones in multiple aspects. The main difference is the extensive sensory setup that combines full-body IMU data with foot pressure and gaze data. In particular, this is the first data set providing natural gait data that includes the visual behavior of the subjects. Another significant difference lies in the trial design. In most data sets, trials aim to capture specific effects such as the influence of the terrain complexity on the gait in an isolated manner. In contrast, our scenarios were designed to capture traits of natural everyday walks, including transitions between various gait patterns. Each trial consists of several minutes of walking in a public space covering common elements such as straight and curvy passages, slopes, stairs, and pavements. All these elements are annotated.

**Table 7:** Overview of publicly available human gait databases.

Name	Subjects	Env.	Sensors	Labels	Trial description	~ Size
MAREA^17^	20 healthy; 33.5 ± 7 years old	indoor, outdoor	4 IMUs (waist, left wrist, both ankles), FSR sensors at sole ends of both feet	HS, TO	treadmill flat/slope with increasing/self-selected speed; walking and running on indoor/outdoor flat space; 1 trial per subject	6 h
Gutenberg Gait Database^43^	350 healthy; 11–64 years old	indoor	Force plate providing GRF and COP data	None	10 m walking; self selected speed; walk on two flat force plates; 20 trials per subject	24 h
HuGaDB^15^	18 healthy; 23.6 ± 3.7 years old	indoor	6 IMUs (both upper legs, both lower legs, both feet), 2 EMGs (both upper leg)	12 activities (walking, sitting, running,…)	self selected speed; varying trial duration for each activity	4 h
Irregular surfaces^18^	30 healthy; 19–33 years old	outdoor	6 IMUs (both upper legs, both lower legs, right wrist, L5/S1 joint)	9 surfaces (flat, grass, cobblestone, stairs up/down, slope up/down,…)	self selected speed; walking on predefined terrain; each trial lasted on average 16 s	7.5 h
Human Locomotion^10^	230 (52 healthy, 53 orthopedic, 125 neurological); 55.5 ± 19.6 years old	indoor	2 IMUs (on both feet)	None	self selected speed; 10 meter walk with turn and walk back; repeated 1–27 times per subject	8.5 h
Gait Analysis Data Base^9^	108 healthy; 17–63 years old	indoor, treadmill	2 IMUs (on both feet), 5 sEMGs (both lower and upper legs, pelvis)	None	cat-walk scenario: walking a distance of 20 m on flat ground at usual, reduced and increased speed, treadmill scenario: walking on a treadmill at incremental speed settings	30 h
Overground and treadmill kinematics^44^	42 healthy; 24 young adults 27.6 ± 4.4 years old, 16 older adults 62.7 ± 8 years old	indoor, treadmill	3D motion capture system with 12 cameras, force plate	None	walking 10 m barefoot on a force plate at usual, reduced and increased walking speed; walking barefoot on a treadmill with 8 different velocities	8.5 h
Ours	20 healthy; 18–69 years old	outdoor	17 IMUs (covering whole body), 8 FSR sensors per foot, eye tracker	9 gait modes (walking, stairs up/down, slope up/down,…) HS, TO	self selected speed; three predefined courses on public places, repeated 3–5 times per subject, each trial lasted between 1–5 min.	9 h

## Technical Validation

The sensors were validated before each recording session in the following way: The Xsens suit was calibrated in the lab before going to the experiment location. The validity of the calibration was checked by visualizing the resulting modeled skeleton in the lab using the Xsens software. The calibration was repeated at the experiment location directly before the recording to account for potential shifts of the IMU sensors. The insoles were validated for each participant by inspecting the pressure signals using the manufacturer’s live-streaming app during a short practice walk of approximately 30 seconds. The eye tracker does not require manual calibration. However, we ensured a reasonable accuracy of the estimated gaze point by letting participants fixate four objects in the vicinity and inspected the estimated gaze point on the world camera video.

## Usage Notes

Each sensor modality is stored in a separate CSV file for each walking task and participant and can be imported into any software framework for further analysis. The labels are also available in separate CSV files. We provide a Python script that generates a single pandas data frame from the CSV files, which can be directly used within standard machine-learning libraries such as Scikit-learn^36^, Pandas^37^, PyTorch^38^ or Tensorflow^39^.

The data provides natural walking behavior annotated by different walking modes and heel strike and toe-off timings. One concrete application could be to train real-time machine learning models on the task of classifying and/or predicting the walk modes and/or hell strike, toe-off timings in order to enhance the control of walk assist systems such as exoskeletons^40^, or prostheses^41^.

## Data Availability

To streamline the processing of the data, we provide various tools and scripts that are accessible at https://github.com/HRI-EU/multi_modal_gait_database. In particular, a Python script is available to join the CSV files into one single pandas data frame, which also supports filtering for specific tasks, participants, and data columns. Furthermore, we provide a visualization tool that jointly displays all three sensor modalities as illustrated by Fig. 6. The tool allows the adjustment of current labels and the creation of custom labels or tags, enabling the generation of additional machine learning tasks.
